# Current-induced viscoelastic topological unwinding of metastable skyrmion strings

**DOI:** 10.1038/s41467-017-01353-2

**Published:** 2017-11-06

**Authors:** Fumitaka Kagawa, Hiroshi Oike, Wataru Koshibae, Akiko Kikkawa, Yoshihiro Okamura, Yasujiro Taguchi, Naoto Nagaosa, Yoshinori Tokura

**Affiliations:** 1 RIKEN Center for Emergent Matter Science (CEMS), Wako, 351-0198 Japan; 2 Department of Applied Physics, The University of Tokyo, Tokyo, 113-8656 Japan

## Abstract

In the MnSi bulk chiral magnet, magnetic skyrmion strings of 17 nm in diameter appear in the form of a lattice, penetrating the sample thickness, 10–1000 μm. Although such a bundle of skyrmion strings may exhibit complex soft-matter-like dynamics when starting to move under the influence of a random pinning potential, the details remain highly elusive. Here, we show that a metastable skyrmion-string lattice is subject to topological unwinding under the application of pulsed currents of 3–5 × 10^6^ A m^–2^ rather than being transported, as evidenced by measurements of the topological Hall effect. The critical current density above which the topological unwinding occurs is larger for a shorter pulse width, reminiscent of the viscoelastic characteristics accompanying the pinning-creep transition observed in domain-wall motion. Numerical simulations reveal that current-induced depinning of already segmented skyrmion strings initiates the topological unwinding. Thus, the skyrmion-string length is an element to consider when studying current-induced motion.

## Abstract (web summary)

Understanding the dynamics of the skyrmion string lattice is the prerequisite for its potential application as next-generation information carriers. Here, the authors explore the topological unwinding of skyrmion string lattice under the application of current pulses.

## Introduction

An ordered solid exhibits elastic (reversible) or plastic (irreversible) deformations, depending on the strength of the force applied. Conceptually similar phenomena have also been observed in electronic systems, such as ferroelectric/ferromagnetic domain walls^1, 2^, flux-line lattices in type-II superconductors^3^, and charge/spin density waves^4, 5^. Domain walls, for example, are more or less meandering by nature in real materials because of random pinning-potential, and they further deform under an effective force, such as a magnetic/electric field or electric current. When the effective force is relatively weak, the induced deformations are small and return to their original positions if the force is removed; that is, domain walls remain trapped in a potential valley (a reversible/pinning regime). In contrast, as the effective force increases, this reversible/pinning regime eventually collapses, and some domain-wall segments begin to exhibit creep, a thermally assisted sluggish motion overcoming potential barriers. Relatively large deformations are thus induced and are no longer reversible (an irreversible/creep regime). This universal behavior is analogous to that in elastic and plastic regimes in ordered solids; therefore, the electronic media described are sometimes viewed as elastic objects.

**Fig. 1:**
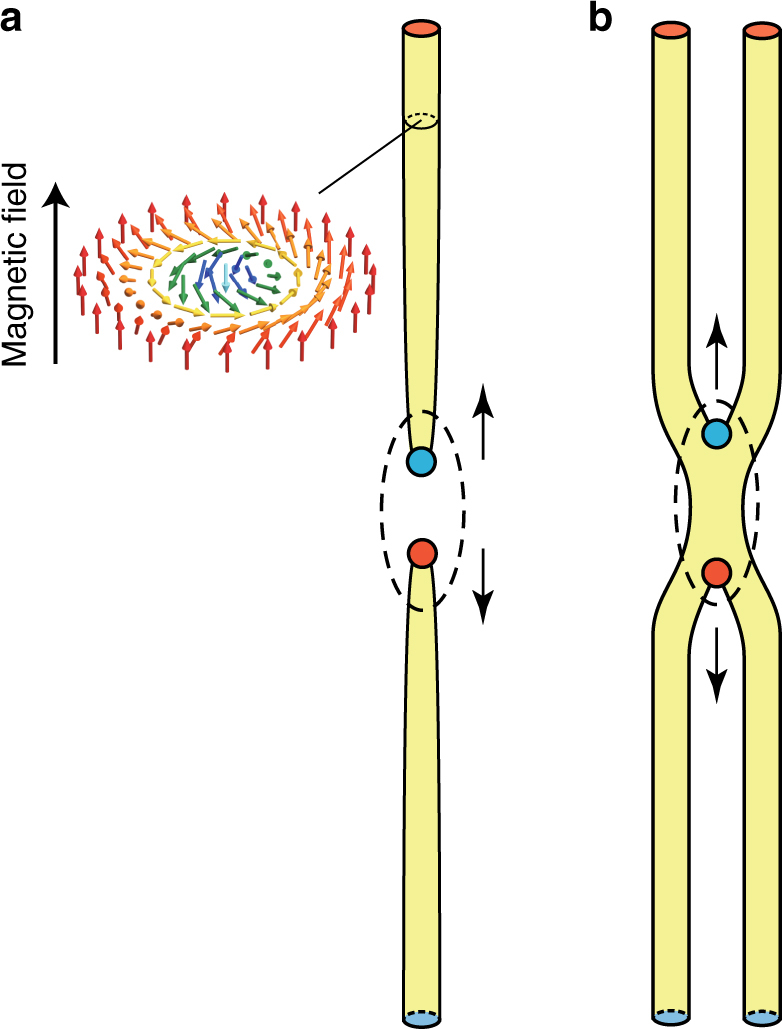
Schematics of the emergent magnetic monopole and antimonopole. **a**, **b** Monopoles/antimonopoles resulting from the pinching-off of a skyrmion string (**a**) and partial merging of two neighboring skyrmion strings (**b**). Colored circles represent emergent magnetic monopoles/antimonopoles. Arrows indicate expected motions of the monopoles/antimonopoles when the topological unwinding progresses

Magnetic skyrmions^6–12^, spin-swirling topological objects of 10–100 nm in diameter, constitute a new member of elastic objects in electronic systems. Whereas the skyrmion is a pancake-like entity in an ultrathin film, it forms a cylindrical structure in a bulk sample^13^ (Fig. 1a), similarly to a flux-line in superconductors. In particular, in bulk chiral magnets, such as MnSi and (Fe_1–x_Co_x_)Si, magnetic skyrmion strings are observed as a thermodynamically stable skyrmion-string-lattice (SkS-L) phase^8, 9^ in which skyrmion strings pointing along an external magnetic field are arranged in the form of a close-packed triangular lattice. Notably, it has been found for MnSi that the thermodynamically stable SkS-L begins to move at extremely low electric current density^14, 15^, on the order of 10^6^ A m^–2^, which is 5–6 orders of magnitude smaller than the value required for current-induced ferromagnetic domain-wall motion^16, 17^. Magnetic skyrmions are therefore attracting considerable research attention as a potential candidate for next-generation information carriers^18–20^.

In current-drive racetrack-type applications^21^ based on isolated skyrmions^22, 23^, one must manipulate a metastable isolated skyrmion because thermodynamically stable skyrmions have a propensity to condense and entirely fill a sample/device^10, 24^; thus, the behavior of metastable isolated pancake-like skyrmions under current is of practical interest. In this context, ferromagnetic ultrathin-film stacks, such as polycrystalline Pt/Co/Ta and single-crystalline Pt/CoFeB/MgO, have recently been investigated^23^, and it has been observed that, whereas almost all skyrmions move uniformly in the single-crystalline Pt/CoFeB/MgO, pinned and moving skyrmions coexist in polycrystalline Pt/Co/Ta. Notably, in the latter case, a moving skyrmion sometimes collides with a pinned one and irreversibly merges into one pinned skyrmion^23^. These observations suggest that under the influence of random pinning potential, current-induced skyrmion dynamics may be more complex than the uniform flow of a fixed number of skyrmions, particularly near the critical current density at which skyrmions begin to move.

The situation may be even more complex in metastable skyrmion strings in a bulk sample. Notably, the diameter of a skyrmion is 10–100 nm, whereas the skyrmion-string length is on the order of the sample thickness (typically, 10–1000 μm). Ordinary bulk crystals at finite temperatures contain a finite density of defects, such as vacancies, as the most thermodynamically stable state, and a defect-free crystal has a higher free energy^25^. Analogously, such a long skyrmion string is unlikely to be intact at finite temperatures but should contain finite topological defects, that is, pairs of the emergent magnetic monopole and antimonopole^26–28^, as schematically shown in Fig. 1a, b. Although the stability and/or dynamics of the emergent magnetic (anti)monopoles are likely directly linked with the controllability of the metastable skyrmion strings, the details remain unclear because of the lack of experiments targeting metastable skyrmion strings in a bulk sample.

In this article, we describe the behavior of the metastable SkS-L in MnSi under pulsed electric current by means of the Hall resistivity, *ρ*_yx_, which is a sensitive probe for condensed topological skyrmions^29, 30^, particularly in MnSi^31–34^. We first write a metastable SkS-L in a limited area of the bar-shaped sample by using a local thermal quenching technique^34–36^ while keeping the other area in the conical state, which is the thermodynamically most stable at the magnetic field investigated; next, *ρ*_yx_ is measured at two distant positions before and after in-plane current pulse applications to determine how the pulse affects the written metastable SkS-L. We find that, whereas the written metastable SkS-L is maintained (the reversible/pinning regime) at low current densities (1–2 × 10^6^ A m^–2^), it undergoes irreversible topological unwinding at higher current densities (>3 × 10^6^ A m^–2^) rather than being transported along the current direction. Moreover, the critical current density above which the topological unwinding appears is larger for a shorter pulse width, reminiscent of viscoelastic characteristics accompanying a pinning-creep transition in domain-wall motion. The smallness of the involved current density and the numerical simulation results suggest that the topological unwinding is initiated by the current-induced depinning of originally segmented skyrmion strings.

## Results

### Sample configuration

**Fig. 2:**
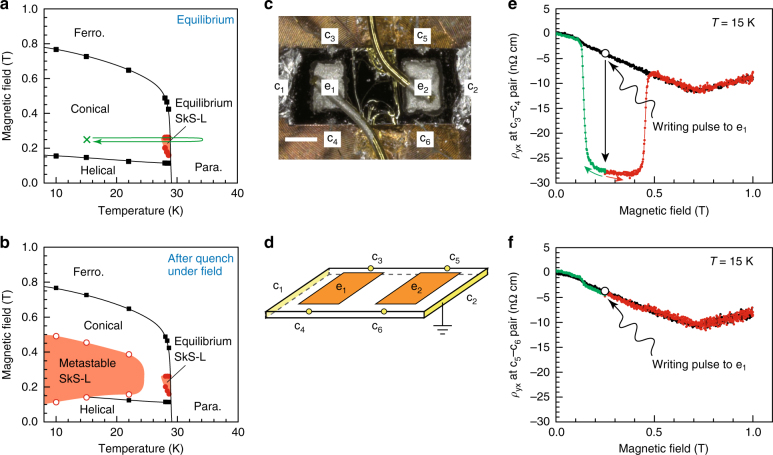
Area-selective writing of the metastable SkS-L in MnSi. **a**, **b** Magnetic state diagrams of MnSi used in this study under equilibrium (**a**) and quenched conditions (**b**). Thermal quenching was performed by applying the writing pulse to the e_1_–c_2_ pair (see **c**, **d**) at 0.249 T; the thermal history that follows the pulse application is schematically shown with the arrow in **a**. The overall features of the phase diagram were reconstructed by referring to the literature^8, 34^, and the magnetic-phase boundaries in the present sample were determined by using a previously reported procedure^34^. **c**, **d** Photograph (**c**) and schematic diagram (**d**) of the sample configuration. The scale bar is 500 μm. **e**, **f** The magnetic-field dependence of *ρ*_yx_ at 15 K, simultaneously measured at the c_3_–c_4_ pair (**e**) and the c_5_–c_6_ pair (**f**). Black curves are the data measured before application of the writing pulse (110 mA and 25 ms) to the electrode e_1_, whereas the red and green ones are those after. The effects of demagnetizing fields are not calibrated

We targeted the archetypal skyrmion-hosting material MnSi and planned to write a metastable SkS-L domain in the matrix of a thermodynamically stable conical order to detect the translation of the SkS-L, if it occurred, by probing the Hall voltage at a distant position. In planning how to prepare the metastable SkS-L in the area of interest, we noted that the SkS-L can be created as a metastable state by employing rapid cooling (>100 K s^−1^) by way of the thermo-equilibrium SkS-L phase (see Fig. 2a, b)^34, 37^. If such thermal quenching is locally implemented, then the metastable SkS-L domain is also expected to be written locally.

Figure 2c, d displays the sample configuration devised for this purpose. In addition to the standard six contacts c_1_–c_6_, two large electrodes e_1_ and e_2_ are attached on the sample surface. The contact resistance of e_1_ and e_2_ is relatively high, ≈10–15 Ω (for comparison, it is less than 0.1 Ω for the current electrodes c_1_ and c_2_), to facilitate local Joule heating; the heating is followed by rapid cooling after the pulse cessation. When a single electric pulse of an appropriate magnitude and width is applied to the e_1_–c_2_ pair (or the e_2_–c_2_ pair), for example, at 15 K and 0.249 T, the SkS-L-to-conical transition line is quickly crossed during such rapid cooling (Fig. 2a), thus leading to the writing of a metastable SkS-L domain around the electrode e_1_ (or e_2_) only. For convenience, we label the area around the electrode e_1_ and e_2_ as areas 1 and 2, respectively, and refer to the electric-heating pulse as a writing pulse.

### Local writing of the metastable SkS-L

The conjectured operation was substantiated by simultaneously probing the Hall voltages near the electrodes e_1_ and e_2_. To this end, we measured *ρ*_yx_–*H* profiles at the c_3_–c_4_ and c_5_–c_6_ pairs at 15 K; these are shown in Fig. 2e, f, respectively. When no writing pulse was applied (the black curves), the c_3_–c_4_ and c_5_–c_6_ pairs exhibited nearly the same profiles, thus reflecting the homogeneous conical order as the initial state. In contrast, after a single writing pulse of 110 mA and 15 ms was applied to the electrode e_1_ at 0.249 T (for the details of the pulse-magnitude dependence, see Supplementary Fig. 1), an enhanced *ρ*_yx_ was observed for the c_3_–c_4_ pair (Fig. 2e), whereas such an enhancement was not discerned for the c_5_–c_6_ pair (Fig. 2f); furthermore, when the magnetic field was subsequently increased (the red curves) or decreased (the green curves) from 0.249 T, *ρ*_yx_ at the c_3_–c_4_ pair remained enhanced and then decreased to the values corresponding to the case in which no writing pulse was applied. This additional Hall signal, Δ*ρ*_yx_ (|Δ*ρ*_yx_|≈24–25 nΩ cm), known as the topological Hall effect^29, 30^, is a hallmark of condensed topological skyrmions^31–34^, thus demonstrating successful writing of the metastable SkS-L domain in the area 1. We also confirmed that when the writing pulse was applied to the electrode e_2_, an enhanced Δ*ρ*_yx_ of a similar magnitude appeared for the c_5_–c_6_ pair, whereas |Δ*ρ*_yx_| ≤ 2 nΩ cm for the c_3_–c_4_ pair (see Supplementary Fig. 2).

### Current application to the metastable SkS-L

Having established a method to create metastable SkS-L in an area-selective manner, we were able to address the type of dynamics that emerges from the metastable SkS-L under current applications. After writing a metastable SkS-L in the area 1, we applied positive in-plane current pulses (that is, current flowing from c_1_ to c_2_) of various magnitudes with a fixed pulse width, 25 ms, and then measured *ρ*_yx_ at the c_3_–c_4_ and c_5_–c_6_ pairs simultaneously to observe the effects of the pulse application.

**Fig. 3:**
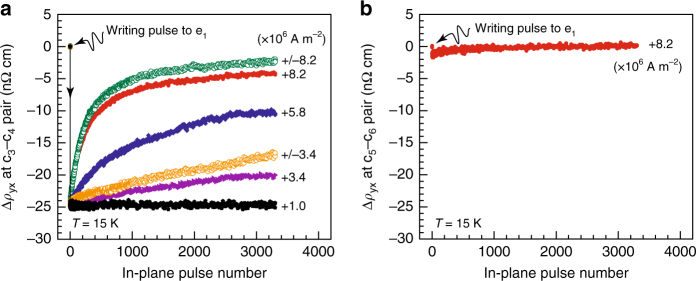
Topological unwinding of the metastable SkS-L under current. **a** Variation in Δ*ρ*_yx_ at the c_3_–c_4_ pair for various pulse magnitudes in response to repeated in-plane pulses. **b** Δ*ρ*_yx_ vs. in-plane pulse number at the c_5_–c_6_ pair for an in-plane current pulse of +8.2 × 10^6^ A m^−2^. Δ*ρ*_yx_, a good measure of the topological Hall signal, is obtained by offsetting the *ρ*_yx_ value before applying the writing pulse to zero. Labels, such as “8.2”, represent the in-plane pulse magnitude used in the measurements, expressed in the unit of 10^6^ A m^−2^. + (closed symbols) denotes the current flowing from c_1_ to c_2_ (see Fig. 2c, d), and +/– (open symbols) denotes the pulse sequence in which positive and negative pulses are applied in an alternating manner. Measurements were performed at 15 K and 0.249 T with a fixed pulse width of 25 ms

Figure 3a, b displays the topological Hall signal, Δ*ρ*_yx_, at the c_3_–c_4_ and c_5_–c_6_ pairs vs. in-plane pulse numbers applied, respectively, for several current magnitudes at 15 K. Here, three aspects can be highlighted. First, at the lowest current density, +1.0 × 10^6^ A m^–2^, the topological Hall signal at the c_3_–c_4_ pair remains constant even after 3000 pulses are applied (Fig. 3a). This finding suggests that the weak current application introduces no appreciable changes to the written metastable SkS-L; that is, at this current density, the metastable SkS-L remains in the reversible/pinning regime. Nevertheless, this regime clearly collapses at higher current densities, and the topological Hall signal (equivalently, the density of skyrmion strings) at the c_3_–c_4_ pair decreases by the repetitive pulse applications. Second, in this irreversible regime, the decrease in the topological Hall signal occurs primarily at the early stage of the repetitive pulse applications; the decrease then becomes more moderate as the pulse number increased (Fig. 3a). Third, no appreciable signal is transferred to the Hall signal at the c_5_–c_6_ pair (Fig. 3b), even after Δ*ρ*_yx_ at the c_3_–c_4_ pair largely disappears. The second and third aspects are not compatible with the simplest scenario that the metastable SkS-L domain may move as a whole along the current direction. In such a case, the Δ*ρ*_yx_ signal at the c_3_–c_4_ pair would remain constant as long as the current-induced shift of the metastable SkS-L were small, and the signal would eventually be transferred to the Hall signal at the c_5_–c_6_ pair; these are not the case in the experiments. We also performed the experiments with negative in-plane current pulses (current flowing from c_2_ to c_1_), but essentially the same results were obtained (Supplementary Fig. 3).

To gain more insight into the decrease in the topological Hall signal, we performed similar experiments but with the alternating application of positive and negative in-plane pulses. In this pulse sequence, the sum of the total effective force applied (including its sign) is zero by definition; thus, the net shift of the metastable SkS-L is not expected, even if each current pulse were to be accompanied by its finite shift. For comparison, the results are plotted in Fig. 3a, labeled with the current magnitude, +/–8.2 and +/–3.4 (×10^6^ A m^–2^). Remarkably, these pulse sequences exhibited a similar (or even larger) decrease in the topological Hall signal. Therefore, the observed Δ*ρ*_yx_ profiles at the c_3_–c_4_ pair cannot be a consequence of the translation of the metastable SkS-L. Alternatively, its destruction can account for this observation: the metastable SkS-L undergoes current-induced topological unwinding into a non-topological spin texture, which is probably the thermodynamically most stable conical state.

Regarding the origin of the topological unwinding, we can rule out a Joule-heating-assisted mechanism. We estimated the increase of the local temperature in the area 1 by measuring the voltage at the c_3_–c_4_ pair during the pulse application and found a temperature increase of only 0.2 K (specifically, 15.0 → 15.2 K) under 8.2 × 10^6^ A m^–2^ and 125 ms (the maximum and longest current density applied in this study: for details, see Supplementary Fig. 4). Moreover, the estimated lifetime of the metastable SkS-L is beyond 10^15^ years at 15 K^34^; thus, the slight temperature increase is expected to play a negligible role in the observed topological unwinding. The appreciable difference of the data between +/–8.2 and +8.2 × 10^6^ A m^–2^ (Fig. 3a) also cannot be ascribed to Joule heating, which should be symmetric with respect to the current polarity, at least, for Ohmic contacts (this is the case in the experiments; see Supplementary Fig. 4).

When considering the destruction of skyrmion strings from the perspective of topology, pair creation of the emergent magnetic monopole-antimonopole (Fig. 1a, b) and their subsequent unbinding motion are expected to be involved^26–28^, analogous to the nucleation and subsequent growth in ordinary first-order phase transitions. To gain insight into which process is more relevant to the current-induced topological unwinding, it is helpful to consider the magnitude of the involved current density. Both a theoretical paper^28^ and our order-of-magnitude estimates (Supplementary Note 1) predict that the monopole-antimonopole pair creation requires a current density of ~10^12^ A m^–2^, orders of magnitude larger than the value used in the present experiments, that is, 10^6^–10^7^ A m^–2^. Therefore, we conclude that the monopole-antimonopole pairs preexist in the quenched SkS-L and hence that their current-induced unbinding motion plays a key role in the topological unwinding. Meanwhile, 10^6^ A m^–2^ is the order on which the skyrmion strings start to move^15^, thus suggesting that the topological unwinding is also related with the creep of the skyrmion strings. In fact, the current-induced topological unwinding at 10 K was found to be less pronounced than that observed at 15 K (Supplementary Fig. 5), in agreement with the notion of creep, which is innately thermally assisted^38–44^.

### Numerical simulations

**Fig. 4:**
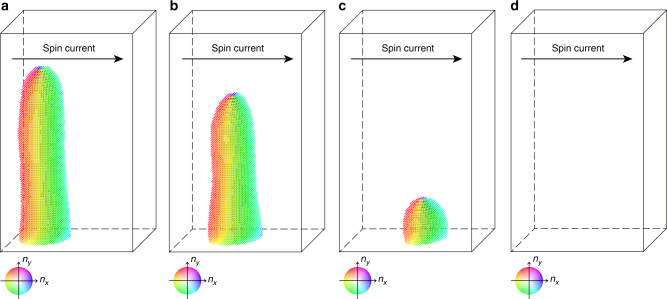
Micromagnetic simulations for a segmented skyrmion string. **a** Initial magnetic state of the simulations, which is metastable under zero current. **b**–**d** Snapshots of the current-induced dynamics of a segmented skyrmion string under *j* = 0.04: *t* = 250 (**b**), *t* = 500 (**c**), and *t* = 650 (**d**) in units of 1/(*γJ*) (see the Methods section). The movie is available in Supplementary Movie 1. Color wheels specify the *x*–*y* plane magnetization direction. The brightness of the color represents the *z* component of the magnetization; that is, the local magnetizations pointing toward the *z* direction are displayed as white. For the details of the calculation, see the Methods section

To determine how the creep of the skyrmion string and the unbinding motion of the preexisting monopole-antimonopole pairs are correlated with each other in the context of the current-induced topological unwinding, we performed micromagnetic simulations of the chiral magnetic system including randomly distributed pinning sites (for details, see the Methods section). We found that under certain numerical conditions, a segmented skyrmion string in a ferromagnetic background can be metastable at zero or weak currents (Fig. 4a). Nevertheless, when the applied current exceeds a specific threshold value, the segmented skyrmion string starts to move; remarkably, this process is also accompanied by the motion of (anti)monopole such that the skyrmion string shortens, as shown in Fig. 4b–d (see also Supplementary Movie 1). As a result, the metastable segmented skyrmion string quickly disappears without a long-distance creep (Fig. 4d). This numerical result establishes a clear example of topological unwinding being initiated by the onset of the creep motion of metastable segmented skyrmion string, corroborating the experimental observations.

### Kinetic nature of the regime transition

We then focused on the kinetic nature of the transition between the reversible/pinning and topological unwinding regimes. In the case of domain walls, an irreversible creep motion occurs more frequently under a larger d.c. force^38–44^. When a pulsed or a.c. external force is applied, however, the pulse width or frequency dictates the onset of creep motion as well: at a given effective-force magnitude, domain-wall segments remain pinned and behave like an elastic solid on short timescales (or at high frequencies), whereas they begin to creep like a viscous fluid on longer time scales (or at low frequencies)^1, 45–48^. Such a dual nature depending on timescale or frequency may be described as a viscoelastic characteristic and can be qualitatively accounted for by considering that deformations exceeding the reversible/pinning regime cannot be induced instantaneously. Although this feature appears to be involved in a wide class of pinned electronic elastic objects, such a viscoelastic nature has not been explored in the skyrmion strings to date.

**Fig. 5:**
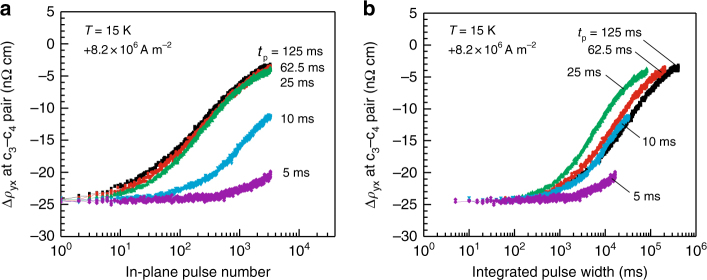
Viscoelastic characteristics of the topological unwinding. **a**, **b** Δ*ρ*_yx_ vs. in-plane pulse number (**a**) and vs. integrated pulse width (**b**) for various pulse width, *t*_p_, plotted on a logarithmic scale. The integrated pulse width represents the pulse number multiplied by each pulse width; the data shown in **a**, **b** are the same data presented in a different manner. Measurements were performed at 15 K and 0.249 T with a fixed pulse magnitude, +8.2 × 10^6^ A m^−2^ (current flowing from c_1_ to c_2_: see Fig. 2c, d)

To address this issue, we investigated the decrease in the topological Hall signal for various pulse widths, *t*_p_, with a sufficiently large pulse magnitude, +8.2 × 10^6^ A m^–2^ (Fig. 5a). As expected, relative to the case of long pulse widths, such as *t*_p_ ≥ 25 ms, much less topological unwinding was observed for the shortest pulse width, 5 ms (for the apparent convergence of the Δ*ρ*_yx_ profiles for *t*_p_ ≥ 25 ms, see Supplementary Note 2). However, it should be emphasized that the low degree of topological unwinding for *t*_p_ = 5 ms occurred not because the integrated pulse width (that is, the pulse number multiplied by each *t*_p_) was the shortest: as illustrated in Fig. 5b, even if the Δ*ρ*_yx_ profiles are compared at a given integrated pulse width, the data for *t*_p_ = 5 ms still exhibits the least topological unwinding. This finding suggests that the metastable SkS-L tends to remain in the reversible/pinning regime for a short-pulse application, whereas it is more likely to enter the topological unwinding regime for a longer-pulse application. This timescale-dependent dynamic transition is analogous to the viscoelastic characteristics accompanying the pinning-creep transition of domain-wall motion^1, 45–48^, again highlighting the key role of the pinning-creep transition of the segmented skyrmion strings at the onset of topological unwinding.

### Pulse width vs. critical current density

The timescale-dependent regime-transition of the metastable SkS-L suggests that the critical current density above which the topological unwinding manifests itself is a function of the pulse width. In estimating the critical current density for various pulse widths, we introduce the quantity *F* (:0 ≤ *F* ≤ 1):

1 $$F(n) \equiv 1 - {\rm{\Delta }}\rho {_{yx}}(n){\rm{/\Delta }}\rho {_{yx}}(0),$$

where Δ*ρ*_yx_(*n*) is a value measured after the positive in-plane current pulse is applied *n* times and Δ*ρ*_yx_(0) denotes a value after applying the writing pulse; thus, *F*(*n*) represents the unwound fraction of the metastable SkS-L after *n*-pulse applications, and *F*(*n*) = 0 and 1 correspond to the as-written SkS-L and fully unwounded non-topological state, respectively.

**Fig. 6:**
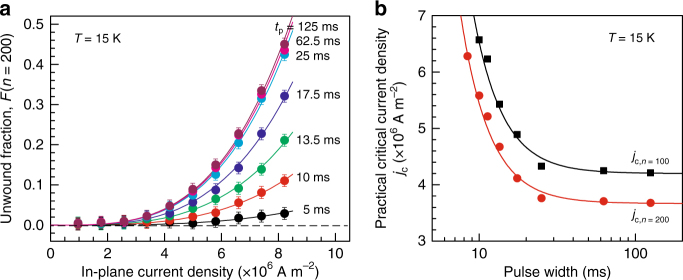
Pulse-width dependence of the critical current density. **a** The topologically unwound fraction of the written metastable SkS-L after the application of 200 in-plane pulses, *F*(*n* = 200), with varying current density, *j*, and pulse width, *t*_p_. The curves are fits to the power low with a fixed exponent, *F*(*n* = 200)~*j* ^3.4^. The error bars are derived from the uncertainty about the measured *ρ*_yx_ values corresponding to ±0.4 nΩ cm. **b** Practical critical current density vs. pulse width. *j*_c,*n* = 100_ and *j*_c,*n* = 200_ denote the current density at which *F*(*n* = 100) and *F*(*n* = 200), respectively, are equal to 0.03 at a given pulse width. Black and red lines are fits to the power law, *j*_c,*n*_ − *j*_c,*n*_(*t*_p_ → ∞)~*t*_p_^–1/*δ*^, with *δ* = 0.43. Measurements were performed at 15 K and 0.249 T

Figure 6a summarizes the results at 15 K after 200 pulse applications, *F*(*n* = 200), as a function of the current density, *j*, for various pulse widths, *t*_p_, showing the clear dependence of *F* on *j* and *t*_p_. In all cases, the *F*(*n* = 200)–*j* profiles (and also the *F*(*n* = 100)–*j* profiles: see Supplementary Fig. 6) are well described by a power law with a fixed exponent, *F*(*n* = 200)~*j* ^*α*^ with *α* = 3.4 (for *F*(*n* = 100), *α* = 3.5: see also Supplementary Fig. 6). This power-law behavior suggests that, in a strict sense, the critical current density is infinitesimally small; however, this consequence is consistent with the current understanding of creep motion, in which an infinitesimal force allows for creep motion with a finite probability as long as the temperature is finite^38–44^.

Nevertheless, we can conditionally derive a critical current density of a practical sense, above which a finite *F*(*n*) becomes experimentally appreciable: in view of the present experimental accuracy, we set this criterion as *F*(*n*) = 0.03 and label the (practical) critical current density with *j*_c,*n*_. Figure 6b displays *j*_c,*n* = 100_ and *j*_c,*n* = 200_ at 15 K as a function of pulse width; although *j*_c,*n* = 200_ is reasonably lower than *j*_c,*n* = 100_ at a given pulse width, a clear correlation between pulse width and critical current density can be seen for the both profiles, thus highlighting the viscoelastic characteristics at the onset of topological unwinding. Although under the present definition, the value of *j*_c_ depends on the pulse number and pulse width, it can be safely concluded that *j*_c_ in the limit of long pulse (*t*_p_ → ∞) is on the order of 10^6^ A m^–2^, in good agreement with the value reported for the thermodynamically stable SkS-L under d.c. currents^14, 15^.

### Power-law analysis of the pulse-width dependence

The *j*_c_–*t*_p_ profiles at 15 K shown in Fig. 6b are both described by a power law with nearly the same exponent: *j*_c_ − *j*_c_(*t*_p_ → ∞)~*t*_p_^–1/*δ*^ with *δ* = 0.43 ± 0.05. The *F*(*n*)–*j* profiles at a lower temperature, 10 K, were also analyzed using power laws, but at relatively large *n* (*n* = 200 and 1000) because of the less pronounced topological unwinding (Supplementary Fig. 6); in this manner, we obtained *α* = 2.4–2.5 and *δ* = 0.50 ± 0.06 at 10 K (Supplementary Fig. 7), to be compared with *α* = 3.4–3.5 and *δ* = 0.43 ± 0.05 at 15 K. Thus, unlike the exponent *α*, the exponent *δ* appears to vary only weakly in the temperature range of 10–15 K. Although there are currently no numerical results to be compared, the value of *δ* would be a potential basis to test the microscopic model for the current-induced dynamics of the metastable SkS-L. At a minimum, the microscopic models for the depinning kinetics have been discussed in charge density waves by comparing the experimentally and theoretically derived *δ* values^49–52^.

## Discussion

We expect that the innately segmented skyrmion strings are likely to be relevant also to the current-induced dynamics of the thermodynamically stable SkS-L. Nevertheless, the consequence of the creep motion is expected to be crucially different between the metastable and thermodynamically stable cases: in a metastable SkS-L, the monopoles and antimonopoles move to eliminate the skyrmion string unless they are pinned, eventually lowering the system’s total free energy. In a thermodynamically stable SkS-L, by contrast, the topological unwinding is not triggered because the breakdown of the thermodynamically stable state inevitably increases the free energy. The current-induced dynamics of the thermodynamically stable SkS-L are therefore expected to involve the steady flow of the monopoles and antimonopoles, which may be accompanied by fluctuations in the emergent electric field^27, 28^.

We have also shown that the existing emergent magnetic (anti)monopoles are harmful when one tries to drive metastable skyrmions with an electric current; therefore, in the context of the skyrmion application, the device thickness must be chosen so that skyrmion strings (or cylinders) do not contain topological defects. In the thermo-equilibrium SkS-L phase, the order of the expected segment length is given as *a* × exp(Δ_MP-AMP_/*k*_B_ *T*), where *a* is the lattice constant of the considered material and Δ_MP-AMP_ is the pair-creation energy of the monopole and antimonopole. According to the numerical results^27^, Δ_MP-AMP_ at the lowest temperature is ≈6 *J* for a simple cubic lattice, where *J* is the magnetic exchange energy and approximately equals to the magnetic transition temperature^53^; hence, for MnSi, Δ_MP-AMP_/*k*_B_ ≈200 K at low temperatures. Thus, in the thermo-equilibrium SkS-L phase (≈27 K), the expected segment length is ~700 nm (or shorter, given that Δ_MP-AMP_ may decrease at high temperatures). Because our sample thickness is ≈100 μm, one can expect that each quenched skyrmion string contains monopole-antimonopole pairs on the order of 100, provided that the topological defect density is frozen at the value of the thermo-equilibrium phase. The direct observation of a segmented skyrmion string remains a challenging issue, which may nevertheless provide useful information when designing a topological-defect-free skyrmion device.

## Methods

### Sample preparation and setup

A single crystal of MnSi was grown via the Czochralski method. The sample was cut and polished to a size of 2.5 × 1.1 × 0.1 mm^3^, with the largest surface normal to the <100> axis. The resistivity ratio *ρ*(300 K)/*ρ*(4.2 K) was ≈52. Gold current leads of 0.3 mm ϕ were attached to the sample and fixed with indium. The electrodes, e_1_ and e_2,_ were constructed using carbon paste to achieve a high contact resistance, ≈10–15 Ω. The sample was mounted on a sapphire substrate in contact with a heat bath and fixed with varnish.

### Transport measurements

The Hall resistivity, *ρ*_yx_, was measured at 33 Hz with a low a.c. current excitation (≈7.97 × 10^4^ A m^–2^) under a magnetic field parallel to the <100> axis using lock-in amplifiers (Stanford Research Systems, SR830) equipped with a transformer preamplifier (Stanford Research Systems, SR554). The Hall resistivity values presented here are antisymmetrized between the positive and negative magnetic fields. The pulse currents that were used to write the metastable SkS-L or to exert an effective force on the written metastable SkS-L were generated by a function generator (NF Corporation, WF1947) connected to a bipolar amplifier (NF Corporation, 4502A). Before applying the writing pulse to the electrode e_1_ at +0.249 T (or –0.249 T), we applied a field of 1T (or –1T) to enter the ferromagnetic state and erase any residue of the metastable SkS-L in the previous measurements. When measuring Δ*ρ*_yx_ as a function of the in-plane pulse number, we waited 1 min after the last pulse was applied to ensure that the sample temperature was sufficiently equilibrated with the sample-holder temperature. When multiple pulses were applied between measurement points, we set the interval between successive two pulses to be 6 s.

### Micromagnetic simulation

The simulation was performed for a simple cubic lattice consisting of 60 × 30 × 100 magnetic moments with an open boundary condition in the *z* direction and periodic boundary conditions in the *x* and *y* directions. We considered the following model Hamiltonian:

2 $$\begin{array}{ccccc}\\ H =- J\mathop {\sum}\limits_{\boldsymbol{r}} {{{\boldsymbol{n}}_{\boldsymbol{r}}} \cdot \left( {{{\boldsymbol{n}}_{{\boldsymbol{r}} + {{\boldsymbol{e}}_x}}} + {{\boldsymbol{n}}_{{\boldsymbol{r}} + {{\boldsymbol{e}}_y}}} + {{\boldsymbol{n}}_{{\boldsymbol{r}} + {{\boldsymbol{e}}_z}}}} \right)} \\ + D\mathop {\sum}\limits_{\boldsymbol{r}} {\left( {{{\boldsymbol{n}}_{\boldsymbol{r}}} \times {{\boldsymbol{n}}_{{\boldsymbol{r}} + {{\boldsymbol{e}}_x}}} \cdot {{\boldsymbol{e}}_x} + {{\boldsymbol{n}}_{\boldsymbol{r}}} \times {{\boldsymbol{n}}_{{\boldsymbol{r}} + {{\boldsymbol{e}}_y}}} \cdot {{\boldsymbol{e}}_y} + {{\boldsymbol{n}}_{\boldsymbol{r}}} \times {{\boldsymbol{n}}_{{\boldsymbol{r}} + {{\boldsymbol{e}}_z}}} \cdot {{\boldsymbol{e}}_z}} \right)} \\ - h\mathop {\sum}\limits_{\boldsymbol{r}} {{n_{\{z,{\boldsymbol{r}}\}}} - {K_{{\rm{imp}}}}} \mathop {\sum}\limits_{{\boldsymbol{r}} \in {{\Lambda }}} {{{({n_{\{z,{\boldsymbol{r}\}}}})}^2},} \\ \end{array}$$

where *J* is the exchange interaction, *D* is the Dzyaloshinskii-Moriya interaction energy, *h*_z_ is the magnetic field along the *z* direction, ***e***_x_ (or ***e***_y_, ***e***_z_) is the unit vector that connects with the nearest neighbor site along the *x* (or *y*, *z*) direction, ***n***_**r**_ is the unit vector of the local magnetic moment at site ***r***, and *n*_{z,**r**}_ is the *z* component of ***n***_**r**_. The last term represents the impurity in the model: the easy axis anisotropy *K*_imp_ was introduced at randomly selected sites, and Λ is the set of random numbers. In simulating the current-induced dynamics of the skyrmion string at zero temperature, we inserted the Hamiltonian into the following Landau–Lifshitz–Gilbert equation:

3 $$\frac{{\mathrm{d}{{\boldsymbol{n}}_{\boldsymbol{r}}}}}{{\mathrm{d}\it t}} = - \gamma \frac{{\mathrm{d}\it H}}{{\mathrm{d}{{\boldsymbol{n}}_{\boldsymbol{r}}}}} \times {{\boldsymbol{n}}_{\boldsymbol{r}}} + \alpha {{\boldsymbol{n}}_{\boldsymbol{r}}} \times \frac{{\mathrm{d}{{\boldsymbol{n}}_{\boldsymbol{r}}}}}{{\mathrm{d}\it t}} - ({\boldsymbol{j}} \cdot \nabla ){{\boldsymbol{n}}_{\boldsymbol{r}}} + \beta \left[ {{{\boldsymbol{n}}_{\boldsymbol{r}}} \times ({\boldsymbol{j}} \cdot \nabla ){{\boldsymbol{n}}_{\boldsymbol{r}}}} \right],$$

where ***j*** represents the (spin-polarized) electric current density. The units of non-dimensional time *t* and current *j* = |***j***| are 1/(*γJ*) and 2*eγJ*/(*pa* ^2^) (*p*: polarization of the magnet), respectively. We chose the following parameter set {*J* = 1.0, *D* = 0.2, *K*_imp_ = 0.2, *h* = 0.018, *α* = *β* = 0.04, *j* = 0.04}. The density of the random impurity was set as 10%.

### Data availability

The data that support the findings of this study are available from the corresponding author on request.

## Electronic supplementary material

Supplementary Information

Descriptions of Additional Supplementary Files

Supplementary Movie 1
