# Supplementary material for: Current-induced viscoelastic topological unwinding of metastable skyrmion strings
Source: Nat Commun. 2017 Nov 6;8:1332. doi: 10.1038/s41467-017-01353-2 (PMC5673897; doi:10.1038/s41467-017-01353-2)
Supplement: Supplementary file 1 — Supplementary Information [file 41467_2017_1353_MOESM1_ESM.pdf]

Supplementary note 1: Order-of-magnitude estimates of the current density required for the monopole-antimonopole pair creation

The creation energy of the monopole and antimonopole pair, $\Delta_{\text{MP-AMP}}$, is approximately given as ≈6J for a simple cubic lattice, where J is the magnetic exchange interaction energy[27]. For the case of MnSi, the magnetic transition temperature (≈ 1.0J: ref. 53) is ~30 K; thus, $\Delta_{\text{MP-AMP}}/k_B$ is estimated to be ~200 K.

Below, on the basis of the Thiele equation, we estimate the order-of-magnitude of the charge current density that is required to compensate $\Delta_{\text{MP-AMP}}$. The Thiele equation for a skyrmion under a steady state is given as follows:

$$4\pi\hat{z} \times (\overrightarrow{j_s} - \overrightarrow{v_\mathrm{d}}) + \kappa\,(\beta\overrightarrow{j_s} - \alpha\overrightarrow{v_\mathrm{d}}) + \vec{F} = 0, \quad (1)$$

where $\overrightarrow{j_s}$ represents the spin-current density, $\overrightarrow{v_\mathrm{d}}$ is the skyrmion drift velocity, $\hat{z}$ is the unit vector along the magnetic field direction, α is the Gilbert damping constant, β is the coefficient of the non-adiabatic effect, $\vec{F}$ is the force acting on the skyrmion, and κ is a constant on the order of unity: all quantities are dimensionless. For simplicity, we consider a situation in which, under a spin current application, a skyrmion segment collides with neighbouring pinned one at a distance of 20 nm (≈ the skyrmion lattice constant of MnSi) in a quasi-static manner (hence, $\overrightarrow{v_\mathrm{d}}$ = 0). Given that β is typically on the order of 10^{-1}–10^{-2} (<< 4π), the Thiele equation reduces to:

$$4\pi\hat{z} \times \overrightarrow{j_s} + \vec{F} = 0 \quad (2)$$

In the present problem, $\vec{F}$ is the repulsive force between the two skyrmions. As long as one assumes that the MP-AMP pair creation occurs when $\Delta_{\text{MP-AMP}}/k_B$ (~200 K) is compensated by the work that the external spin current has done against the repulsive force, the required force can be estimated to be on the order of 10^{-13} N (= $|\vec{F}|J/a$, where $|\vec{F}|$ is the dimensionless force in the Thiele equation and a is the lattice constant for MnSi, ≈4.5 Å). Thus, the required charge current, $\overrightarrow{j_\mathrm{e}}$ [A/m^2], is estimated to be 10^{12} A/m^2, where $\overrightarrow{j_\mathrm{e}}$ is given as $\overrightarrow{j_s}\times 2e\gamma J/(pa^2)$ with p (= 0.2) being the polarization of the magnet and γ being the gyromagnetic ratio. The estimated value is also consistent with that reported in a theoretical paper[28].

Supplementary note 2: The apparent convergence of the $\Delta\rho_{yx}$ profiles for $t_p \geq 25$ ms

In Fig. 5a, the $\Delta\rho_{yx}$ profiles roughly converge for pulse widths longer than 25 ms. This observation has two implications. First, when a single current pulse is considered, the aggregate of skyrmion strings eventually reach a steady state under the current application; consequently, further duration of the single current pulse introduces little change to the aggregate, in which some fraction is already relaxed into a non-topological state at the early stage of the pulse duration. Second, because the topological unwinding nevertheless progresses when the next pulse is applied, the partly relaxed aggregate of skyrmion strings appears to be rearranged during a pulse interval and to result in a skyrmion-string aggregate that is distinct from an aggregate immediately before the pulse cessation; such an updated aggregate can further relax if a subsequent pulse is applied. Nevertheless, we note that these implications remain largely speculative.

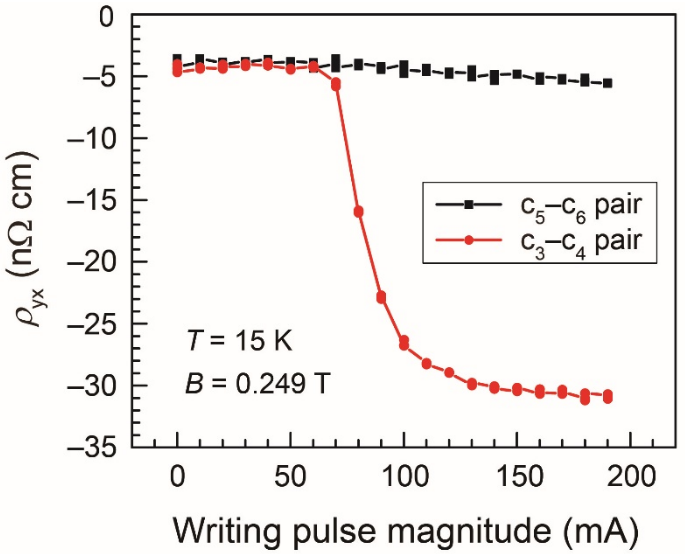

Supplementary Fig. 1 | ρ_{yx} at the c_3–c_4 and c_5–c_6 pairs versus the writing pulse magnitude applied to the electrode e_1. Measurements were performed at 15 K and 0.249 T with a fixed pulse width of 15 ms. When the pulse-current magnitude is weak, ρ_{yx} at the c_3–c_4 and c_5–c_6 pairs exhibit nearly the same value, thus indicating the homogeneous conical order. In contrast, when the pulse-current magnitude is sufficiently large, an enhanced ρ_{yx} is observed for the c_3–c_4 pair, whereas such an enhancement is not discerned for the c_5–c_6 pair.

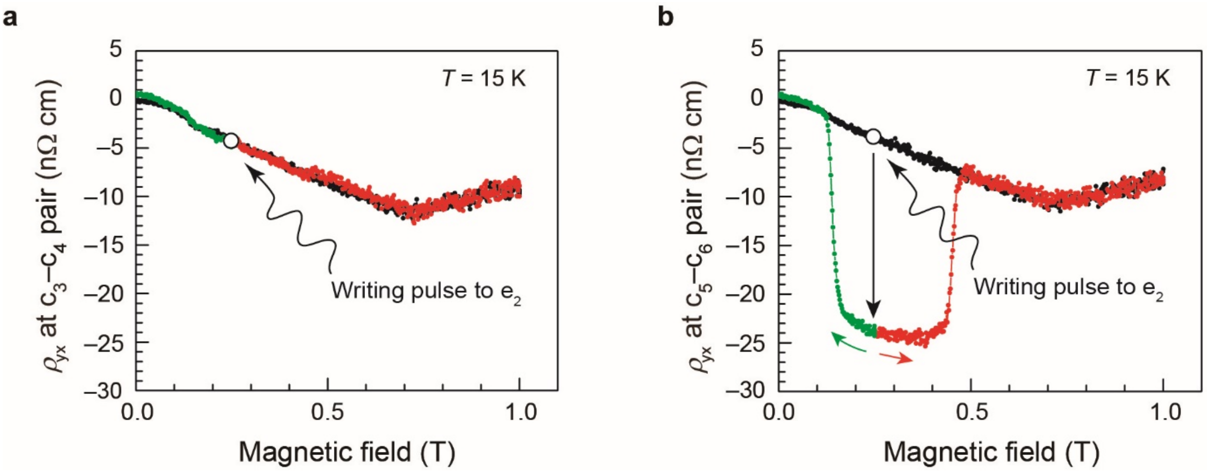

Supplementary Fig. 2 | Magnetic-field dependence of ρ_{yx} at 15 K, simultaneously measured at the c_3–c_4 pair (a) and the c_5–c_6 pair (b). Black curves are the data measured before application of the writing pulse (150 mA and 15 ms) to the electrode e_2, whereas the red and green ones are those after.

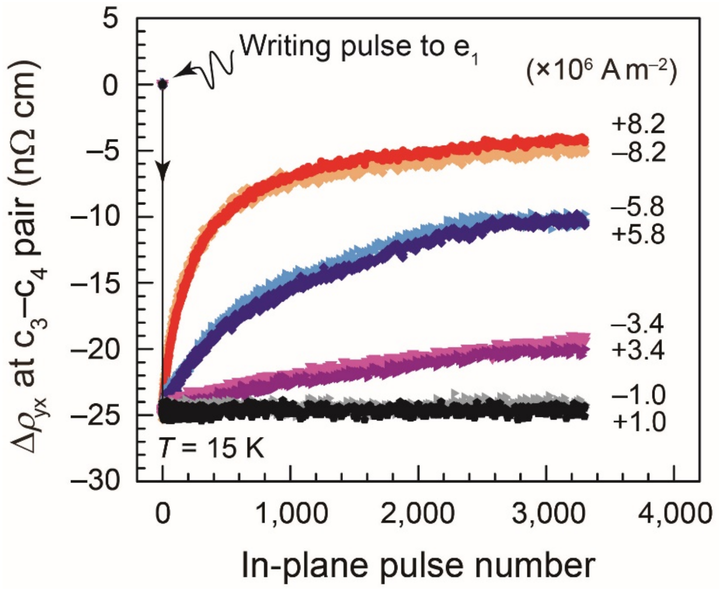

Supplementary Fig. 3 | Topological unwinding of the metastable SkS-L under positive and negative current-pulse applications. Labels, such as "8.2", represent the in-plane pulse magnitude used in the measurements, expressed in the unit of 10^6 A m^{-2}. + and – denote current flowing from c_1 to c_2 and c_2 to c_1, respectively (see Figs. 2c,d). The data for the positive current are the same with those shown in Fig. 3a. Measurements were performed at 15 K and 0.249 T with a fixed pulse width of 25 ms.

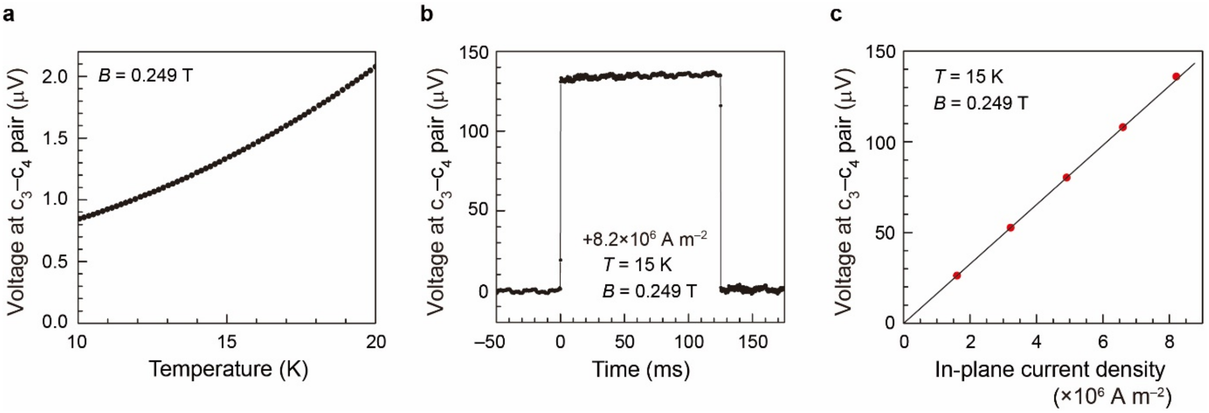

Supplementary Fig. 4 | Estimation of local temperature increase due to Joule heating. a, The temperature dependence of voltage at the c_3–c_4 pair measured with 7.97×10^4 A m^{-2}. Because of a slight misalignment of the contacts c_3 and c_4, the raw voltage data at the c_3–c_4 pair are dominated by the longitudinal resistivity, ρ_{xx}, unless the measured voltage is antisymmetrized with respect to positive and negative magnetic fields. This voltage-temperature profile can be used as a suitable reference when estimating a local temperature of the area 1. **b,** Time profile of voltage at the c_3–c_4 pair during the in-plane pulse application of 8.2×10^6 A m^{-2} with 125 ms width. **c,** Current-voltage characteristics at the c_3–c_4 pair. The voltages are derived by measuring the voltage-time profile for each pulse magnitude, as shown in **b**. At the largest pulse magnitude, 8.2×10^6 A m^{-2}, the slight non-linearity of ≈2 % is appreciable in the current-voltage characteristics, which can be ascribed to a temperature increase of 0.2 K by referring to the voltage-temperature profile shown in **a**.

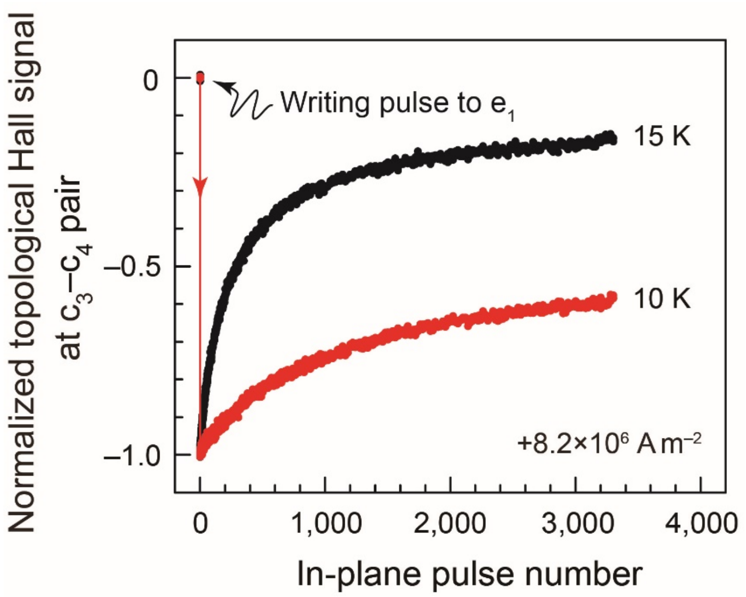

Supplementary Fig. 5 | Comparison of the degree of topological unwinding between 10 and 15 K. Because the topological Hall signal is intrinsically temperature dependent (see ref. 34), the normalized topological Hall signals are displayed, thus facilitating a fair comparison of the degree of topological unwinding between different temperatures. Here, –1 and 0 in the vertical axis correspond to the as-written metastable SkS-L and fully non-topological conical state, respectively. The measurements were performed at 0.249 T with a fixed pulse magnitude of $+8.2\times10^{6}$ A m^{-2} and a pulse width of 25 ms.

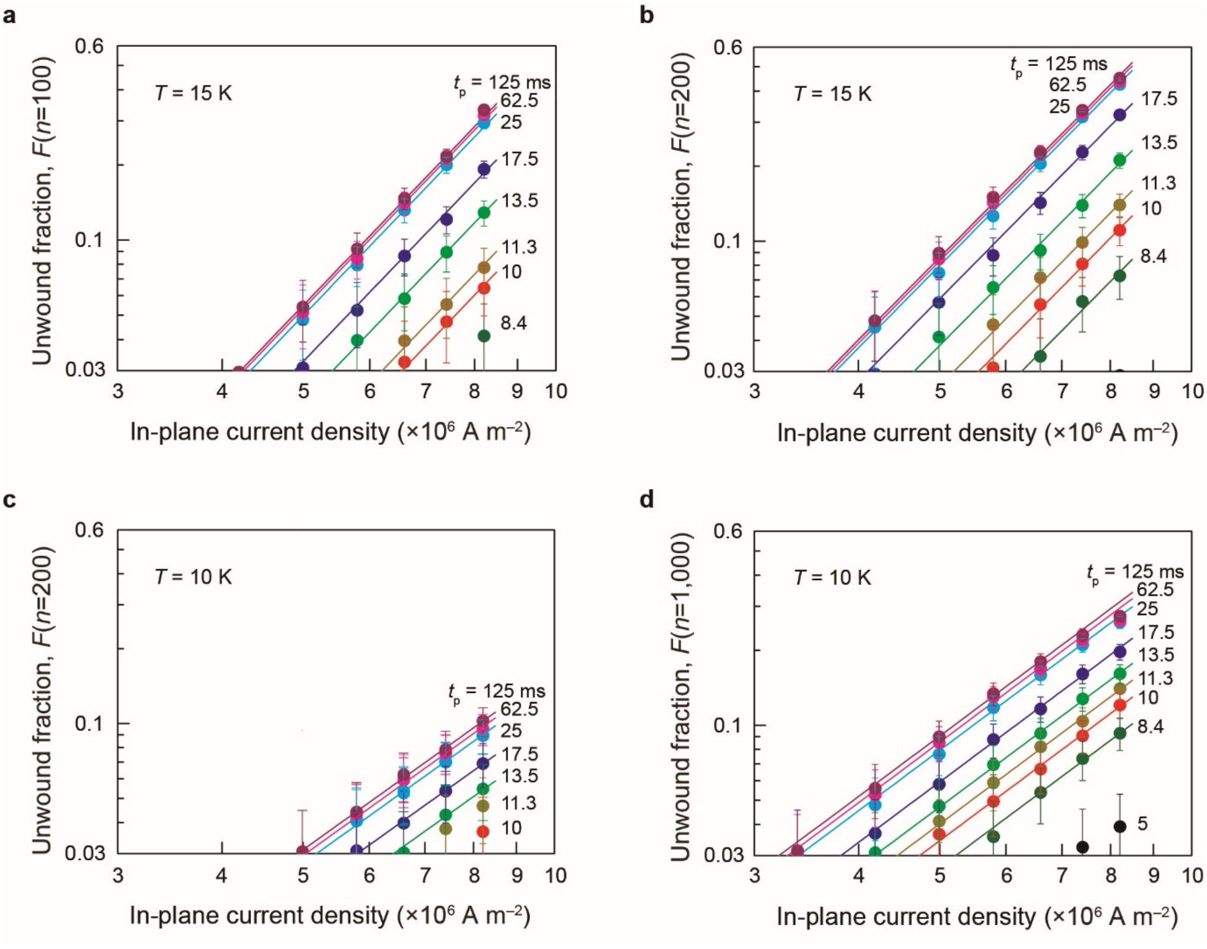

Supplementary Fig. 6 | Topologically unwound fraction of the metastable SkS-L after application of in-plane pulses *n* times, *F*(*n*), for various values of the current density, *J*, and the pulse width, t_p. a,b, *F*(*n* = 100) (**a**) and *F*(*n* = 200) (**b**) at 15 K. Lines are fits to the power law, $F \sim J^{\alpha}$ with α = 3.5 for *F*(*n* = 100) and α = 3.4 for *F*(*n* = 200). **c,d,** *F*(*n* = 200) (**c**) and *F*(*n* = 1,000) (**d**) at 10 K. Lines are fits to the power law, $F \sim J^{\alpha}$ with α = 2.4 for *F*(*n* = 200) and α = 2.5 for *F*(*n* = 1,000). Error bars represent the measurement uncertainty corresponding to ±0.4 nΩ cm. Measurements were performed at 0.249 T.

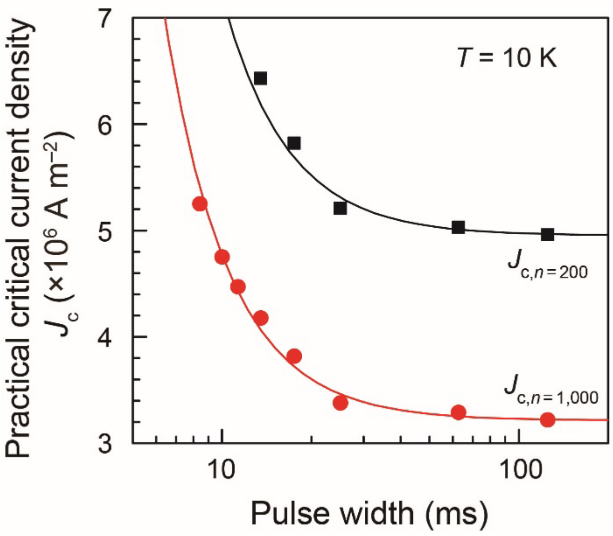

Supplementary Fig. 7 | Practical critical current density versus pulse width at 10 K. $J_{c,n\,=\,200}$ and $J_{c,n\,=\,1,000}$ denote the current density at which $F(n = 200)$ and $F(n = 1,000)$, respectively, are equal to 0.03 at a given pulse width. Black and red lines are fits to the power law, $J_{c,n} - J_{c,n}(t_p \rightarrow \infty) \sim t_p^{-1/\delta}$, with $\delta = 0.50$. Measurements were performed at 0.249 T.
