# Supplementary material for: Current-induced viscoelastic topological unwinding of metastable skyrmion strings
Source: Nat Commun. 2017 Nov 6;8:1332. doi: 10.1038/s41467-017-01353-2 (PMC5673897; doi:10.1038/s41467-017-01353-2)
Supplement: Supplementary file 2 — Descriptions of Additional Supplementary Files [file 41467_2017_1353_MOESM2_ESM.pdf]

Description of Additional Supplementary Files

File Name: Supplementary Movie 1
Description: Micromagnetic simulations for the current-induced dynamics of a segmented skyrmion string. The used parameters are shown at the bottom (see also the Methods section). The density of the random impurity was set as 10 %. Selected snapshots are displayed as Fig. 4 in the main text.
